#  IntNetDB v1.0: an integrated protein-protein interaction network database generated by a probabilistic model

**DOI:** 10.1186/1471-2105-7-508

**Published:** 2006-11-18

**Authors:** Kai Xia, Dong Dong, Jing-Dong J Han

**Affiliations:** 1 Chinese Academy of Sciences Key Laboratory of Developmental Biology, Center for Molecular Systems Biology, Institute of Genetics and Developmental Biology, Chinese Academy of Sciences, Datun Road, Beijing, 100101, China; 2 Graduate University of Chinese Academy of Sciences, Beijing, 100049, China

## Abstract

**Background:**

Although protein-protein interaction (PPI) networks have been explored by various experimental methods, the maps so built are still limited in coverage and accuracy. To further expand the PPI network and to extract more accurate information from existing maps, studies have been carried out to integrate various types of functional relationship data. A frequently updated database of computationally analyzed potential PPIs to provide biological researchers with rapid and easy access to analyze original data as a biological network is still lacking.

**Results:**

By applying a probabilistic model, we integrated 27 heterogeneous genomic, proteomic and functional annotation datasets to predict PPI networks in human. In addition to previously studied data types, we show that phenotypic distances and genetic interactions can also be integrated to predict PPIs. We further built an easy-to-use, updatable integrated PPI database, the Integrated Network Database (IntNetDB) online, to provide automatic prediction and visualization of PPI network among genes of interest. The networks can be visualized in SVG (Scalable Vector Graphics) format for zooming in or out. IntNetDB also provides a tool to extract topologically highly connected network neighborhoods from a specific network for further exploration and research. Using the MCODE (Molecular Complex Detections) algorithm, 190 such neighborhoods were detected among all the predicted interactions. The predicted PPIs can also be mapped to worm, fly and mouse interologs.

**Conclusion:**

IntNetDB includes 180,010 predicted protein-protein interactions among 9,901 human proteins and represents a useful resource for the research community. Our study has increased prediction coverage by five-fold. IntNetDB also provides easy-to-use network visualization and analysis tools that allow biological researchers unfamiliar with computational biology to access and analyze data over the internet. The web interface of IntNetDB is freely accessible at . Visualization requires Mozilla version 1.8 (or higher) or Internet Explorer with installation of SVGviewer.

## Background

Protein-protein interactions (PPIs) underlie most biological processes. Dissecting the PPI network for a particular biological process may provide important clues into molecular mechanisms of the process [1]. Recently, large-scale experimental studies have generated many PPI datasets in different model organisms by yeast two-hybrid (Y2H) screens [2-8] and by co-affinity purification (co-AP) followed by mass spectrometry (MS) [9,10]. These studies have provided opportunities to examine cellular function at a network level.

There are two shortcomings of these data: (a) the coverage is very low and far from complete, and (b) the accuracy of each dataset is generally not very high and varies considerably from dataset to dataset [11]. The unreliability and incompleteness of PPI data complicates elucidation of biological processes or cellular functions, and may potentially misrepresent the topological features of the network [12]. Many methods have been used to predict PPI networks [13]. These fit into three categories: sequence based [14], high-throughput data-based, and a combination of sequence and high-throughput data. The sequence-based prediction methods include gene fusion, gene neighborhood and phylogenic profiles [15], and predictions based on protein/domain structure [16,17]. The high-throughput data based methods predict PPIs from data generated by high-throughput experiments, such as correlated mRNA expression [11,18], correlated phenotype profiles [19], shared protein interaction partners [20], shared genetic interaction profiles [21,22], or similar subcellular localizations [17]. The combination methods predict interologs based on gene orthologs [23,24].

Recently machine learning methods have been introduced to predict PPIs by combining genomic and experimental features. Bayesian classifiers are probability-based and competent in integrating large numbers of heterogeneous datasets [25-27]. Probabilistic decision trees and random forest (a collection of decision trees) specialize in classifying objects into different categories [28-31]. Logistic regression is especially suited for assigning elements into two opposing groups [32-35]. Support vector machines (SVM) have been used to predict PPIs from a limited number of attributes to binary outputs (interact versus not interact), but has not been used for integrating multiple evidences [36-43].

Among these machine learning approaches, Bayesian probabilistic model has many unique advantages in predicting PPIs. It can handle heterogeneous data types, such as numerical phenotype values, discrete survival fitness values, vector microarray expression values, binary interactome values or categorical Gene Ontology annotation values. Heterogeneous data types can be transformed into one uniform probabilistic score by calculating the likelihood ratios. Each data source is automatically weighted according to its confidence level. Missing data are tolerable for integration. Furthermore, Bayesian model is a fast simple algorithm, as it is probability-based and does not require much time to standardize different data of different sources or types. Most importantly, Bayesian model has been proven by previous studies to be particularly competent in predicting PPIs [31,32]. Lastly, the simple integration scheme is very suitable for updating or including future datasets.

To date the Bayesian model has mostly been applied to yeast, and rarely to predict human PPI [27,44]. Rhodes et al integrated 13 datasets of four different data types: physical interactions in model organism, co-expression, domain-domain interactions and shared biological functions [27]. However, other types of high-throughput data then available were not examined. Since the publication of this analysis many other high-throughput data have been generated, some directly done on human proteins. Furthermore, the ever-growing high-throughput data and the data mining demand from the research community require a more comprehensive, current and updatable integration platform and database for integrating, storing, visualizing and mining the data. Toward achieving these goals, we examined the predictive power of new data types and datasets, created an Integrated Network Database (IntNetDB) and provided easy-to-use web-based visualization and data mining options.

We chose to adopt the Bayesian analysis method, because of unique advantages in predicting PPIs [45], and because of its proven effectiveness established by previous studies [25-27]. From the first [25] to the latest study [27] using this analysis framework, more accurate and more extensive integrated PPI networks have been predicted. Here, using ten-fold cross validation, we also demonstrated the effectiveness of Bayesian analysis in predicting human PPIs from 27 datasets of seven different data types.

**Figure 1:**
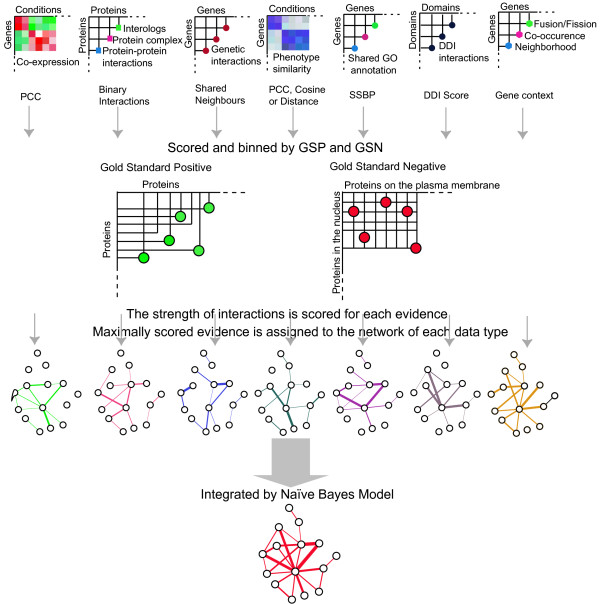
**A brief description of the integration by the probabilistic model**. Seven heterogeneous dataset types are gathered and evaluated by the gold standard positive (GSP, all the annotated protein-protein interactions from HPRD) and gold standard negative (GSN, possible protein pairs between the proteins on the plasma membrane and those in the nucleus). The potential of forming a protein-protein interaction is scored as the likelihood ratio (LR) for protein pairs to be true positive interactions versus true negative interactions, according to the GSP and GSN. Each interaction is assigned a LR within a data type. When evidence arises from more than one datasets within a data type, the maximal LR among the datasets is used for a gene pair. Then the LRs given by different data types are integrated by the Naïve Bayes model, which generates the final prediction score for a potential PPI by multiplying all the LRs from the seven distinct data types. Lastly, the final integrated network with an acceptable confidence level for each interaction is presented.

To allow researchers easy and rapid use of our prediction results, we assembled the data in a web-accessible Integrated Network Database, and we provide a graphic-user-interface for querying PPIs among a group of query proteins/genes (Figure 1). We also provide an online tool for creating customizable visualization of the network, and a computational method to search for highly-connected network modules, as these modules frequently correspond to molecular machines [19]. We used the MCODE algorithm with default settings to find network modules based on network topology. The details about the algorithm and the principles are in the original paper [46]. Furthermore, the design of the database and user interface allows easy incorporation of new datasets.

## Construction and content

### Gold standard for integration

Naïve Bayes classifiers require a gold standard positive (GSP) and a gold standard negative (GSN) dataset. The Human Protein Reference Database (HPRD) [47] is a protein-protein interaction database with 19,438 distinct interactions among 5,983 proteins. It is manually curated by expert biologists based on small-scale and focused experiments described in the scientific literature. We accepted it as high quality and used it as the GSP dataset. We used the GSN dataset previously generated by Rhodes et al [27], which includes all the possible pair-wise combinations between two sets of proteins that are assigned a subcellular localization of the plasma membrane (1397 proteins) and the nucleus (2224 proteins), respectively, by the Gene Ontology (GO) Consortium [48]. The GSN includes a total of 3,106,928 interactions. The size and the ratio of GSP and GSN are adequate for covering interactions of low prediction probability and for predicting human PPIs [27]. To measure the predictive power or confidence level, we used the likelihood ratio (LR) of a gene pair to be a true positive interaction versus a true negative. This is calculated by Pr(E|GSP)/Pr(E|GSN), where the Pr(E|GSP) is the probability of a certain evidence observed within GSP set and Pr (E|GSN) is the probability of a certain evidence observed within GSN set (**Methods**).

### Physical protein-protein interactions

**Figure 2:**
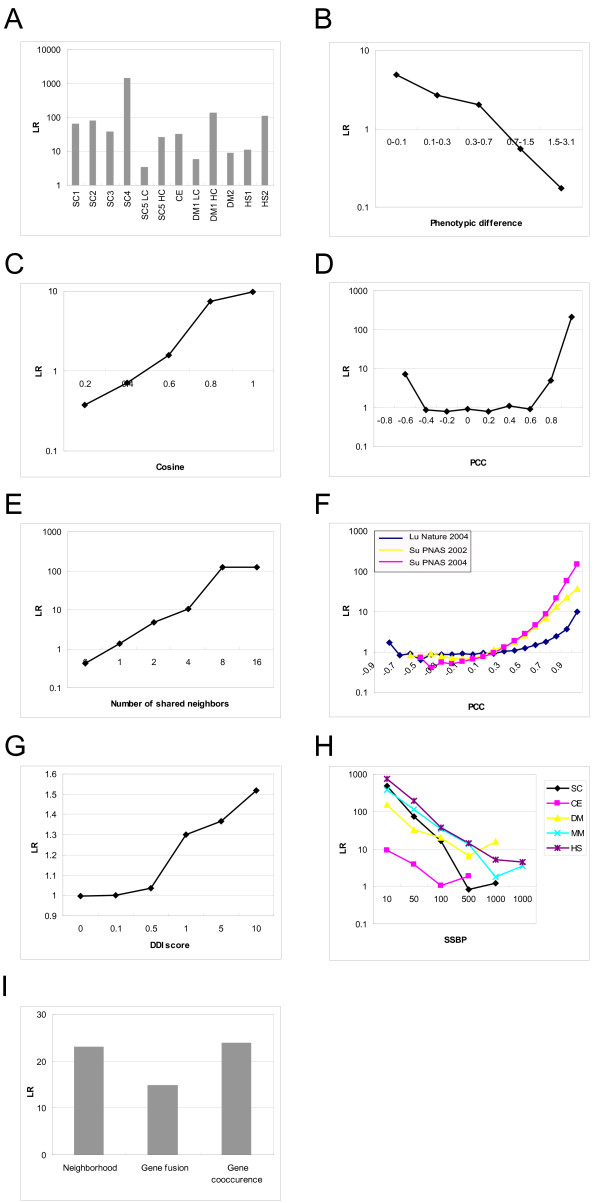
**Assessing the performance of each dataset in predicting the human protein-protein interactions**. A. Large-scale protein-protein interaction (PPI) datasets from model organisms and human. The datasets SC5 and DM1 are binned by their confidence level given by the original studies: LC, low confidence; HC, high confidence. B-D. Phenotypic datasets from model organisms. The fly genome-wide RNAi dataset is evaluated by the arithmetic difference in phenotypic values between a pair of genes (D). The phenotypic similarity of yeast genes upon knock-out is evaluated by cosine distance for the discrete values (C) and PCC for the continuous values (D). E. Yeast genetic interaction datasets. Yeast genetic interactions are grouped by the number of shared neighbors between a pair of genetically interacting genes. F. Large-scale human gene expression datasets. Gene pairs are binned by their Pearson Correlation Coefficient (PCC) between the expression profiles of the pair. The purple, yellow and blue curves are derived from three different expression datasets [57-59]. G. Domain-domain interaction (DDI) score. The DDI score of a domain pair is assigned to a pair of proteins containing the domains. If different scores exist between a pair of proteins arising from different interacting domain pairs, the maximum of the scores is assigned to the pair. The protein pairs are grouped according to their DDI scores. H. Smallest number of shared biological processes (SSBP) of yeast (SC), worm (CE), fruitfly (DM), mouse (MM) and human GO annotations. Gene pairs are binned by the smallest number of shared GO annotations between a pair of genes. Then the LR of being GSP versus GSN is calculated and plotted for gene pairs within each bin for each organism. I. Gene context analysis to predict PPIs. Three types of *in silico *prediction results are evaluated (gene fusion, gene co-occurrence and gene neighborhood).

As PPIs are frequently conserved through evolution [49,50], we gathered the high-throughput experimental PPI data of the model organisms *Saccharomyces cerevisiae *[2,3,9,10,51] (SC1-5, yeast interactome dataset 1–5 [2,3,9,10,51]), *Caenorhabditis elegans *[5] (CE), and *Drosophila melanogaster *[4,6] (DM1 and DM2, fly interactome dataset 1 and 2 [4,6]). We mapped interactome data from these different model organisms to human through protein orthologs determined by best reciprocal BLASTP hit (**Methods**). The confidence for each of the datasets was evaluated. Whenever confidence level information is available in the original study, we divided the dataset into different groups according to their confidence levels (Figure 2A). The results indicate that interologs (ortholog pair) derived by these methods generally have strong predictive power. Among them the *S. cerevisiae *interactome dataset 4 [10] is the most predictive (LR = 1438.9). We also evaluated the recently published human interactome [7,8] (HS1 and HS2, human interactome dataset 1 [7] and dataset 2 [8]) and calculated the LR for those two datasets (LR1 = 11.1 and LR2 = 112.5). In brief, all the physical interaction datasets examined can be incorporated in the integration (Figure 2). The big differences in predictive power of different datasets (even those generated by the same method over the same proteome) point to the necessity of data annotation and integration before utilizing the data to derive biological hypotheses.

### Phenotypic data from model organisms

Loss of function among interacting or functionally related proteins tends to result in similar phenotypes [52-54]. Several large-scale phenotypic datasets are available for model organisms [52-54]. RNAi phenotype data have been used to predict PPIs for model organisms [19]. To examine whether phenotype data from model organisms are also predictive for human PPIs, we transferred phenotype data from model organisms to human by matching the genes in model organisms to their corresponding human orthologs. We then calculated the pair-wise phenotype similarity scores between genes. Considering the various forms of the phenotypic data, we used different measurements for phenotypic distance depending on the form of phenotypic values. For the dataset with one value for each gene under a single condition, we simply used the absolute value of arithmetic difference between the phenotypic values [52]. For the dataset with discrete values under multiple conditions, we used the cosine value between the phenotypic values of a pair of genes [54]. For the dataset with continuous value under multiple conditions [53], the Pearson Correlation Coefficient (PCC) was used. Then each phenotypic dataset was binned according to the similarity score (phenotypic difference, cosine or PCC) and the LRs were evaluated within each bin. A correlation between phenotypic similarity and LR can be observed even in the cross-species phenotypic datasets (Figure 2B–D). Therefore phenotype data can also be integrated to predict the PPI network.

### Genetic interactions from model organisms

Synthetic genetic analysis (SGA) has been used in *Saccharomyces cerevisiae *[21,22] to globally map yeast genetic interactions. A significant overlap between PPIs and genetic interactions was recently demonstrated [55]. The number of common neighbors between a pair of genetically interacting genes can be used to predict potential PPIs [22]. We implemented such an analysis on binary yeast genetic interaction datasets [21,22]. First, the genetic interactions were mapped as human interologs. Then we binned all the interactions by the number of the shared neighbors, and the LRs were calculated for each bin (Figure 2E). We found that the more neighbors a pair of genetically interacting genes share, the more likely a direct PPI occurs between them. These genetic interaction data actually gave rise to very high confidence PPI predictions, slightly lower than large-scale PPI mapping (Figure 2E).

### Gene co-expression

Genes that exhibit mRNA co-expression tend to show protein interaction [56], especially for those in the same complex or in the same biochemical reaction. Such correlated genes might be regulated by the same transcriptional factor or a set of transcription factors. We examined three high quality large-scale microarray datasets [57-59] to predict the human PPIs. One dataset consists of gene expression profiles during aging of the human brain, and the other two consist of gene expression profiles across a variety of tissues or cell lines. For each dataset, gene pairs were assigned into 20 bins of increasing pairwise expression PCC values (Figure 2F). We observed a significant correlation between expression PCC and the likelihood of forming direct PPI (measured by LR) between a pair of genes when the PCC is above 0.5, which may help to predict human PPIs (Figure 2F).

### Shared functional annotation

Proteins with the same biological function are more likely to physically interact than those without. In addition, proteins sharing a more specific annotation are more likely to interact than those sharing a commoner less specific annotation. The Gene Ontology Consortium [48] has assigned 4,416 GO annotations to 14,801 human genes (proteins). To quantify the similarity between gene annotations, we identified the smallest shared biological process (SSBP) between a pair of genes [27]. The SSBP is calculated by three steps: (1) find all the GO terms shared by each pair of genes, (2) find the number of other genes also sharing these GO terms, (3) get the GO term with the smallest gene count. In agreement with expectation, the smaller the SSBP count, the more likely the proteins are to directly interact (Figure 2G). We also examined the GO annotations of four model organisms (*Saccharomyces cerevisiae, Caenorhabditis elegans, Drosophila melanogaster *and *Mus musculus*). When genes from the four model organisms were mapped to human orthologs, the SSBP still has good predictive power to predict human PPIs (Figure 2G).

### Domain-domain interaction (DDI)

Many protein-protein interactions are mediated by domain-domain interactions. If two domains can physically interact, proteins containing these two domains are also likely to interact. DDI predictions have been explored before. We used the domain-domain interaction scores in InterDom, which are derived largely from structural information [16]. After transferring the scores to each pair of proteins that contain the interacting domains, we observed a weak but still clear correlation of the domain interaction score with LR (Figure 2H).

### Gene context analysis

Gene context refers to *in silico *PPI predictions based on genome sequences [11]. Three types of gene context information have been used to predict protein-protein interactions: a) gene fusion/fission, which finds that interacting proteins in one species are more likely to be fused into one single protein in another species; b) gene co-occurrence which finds that interacting proteins are more likely to be found both present or both absent in an organism with a fully sequenced genome; c) gene neighborhood which finds that functionally coupled genes (interacting proteins or proteins in a complex) are more likely to be located in the same operon or gene cluster in a genome. Gene context analysis has been previously performed by von Mering *et al*, [11] to generate *in silico *PPIs for *Saccharomyces cerevisiae*. We mapped these interactions to human through interologs and found that all three types of *in silico *PPI predictions are suitable for predicting human PPIs (Figure 2I).

### Integration by probabilistic model

We chose the Naïve Bayes classifier model because it integrates all the independent evidences simply by multiplying the LRs of diverse evidences [25]. Such expediency will facilitate integration of more datasets in the future. Using such an integration scheme, many weak evidences from several data types can be accumulated to predict interactions with increased confidence. We assume that different data types are conditionally independent, because they are derived from different experimental technologies that aim to measure different biological features of genes/proteins or protein pairs, or are from unrelated annotations. We categorized the 27 datasets into seven different types (Figure 1) to satisfy the requirement of conditional independency by the Naïve Bayes classifier. The LR contributed by each dataset toward the predicted confidence score of a gene pair (Figure 1) was set to the LR observed for the corresponding dataset bin where the gene pair was found (Figure 2). Then the maximal LR among all the datasets of the same data type was chosen to represent the LR contributed by the particular data type toward the prediction score. Finally the ratios determined by different data types were multiplied to represent the combined LR [25,27]. Base 2 logarithmic form of LRs (LLRs) was used to calculate the final score.

### Cross validation of the integration results

**Figure 3:**
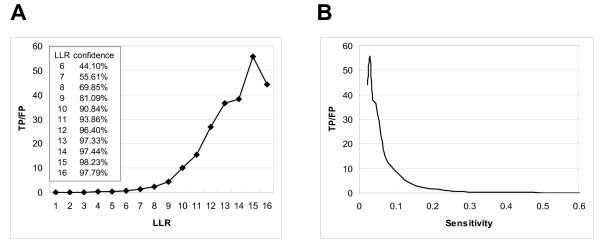
**TP/FP ratios (true positive versus false positive) at different LLR cutoffs or sensitivity by 10-fold cross-validation**. The TP/FP ratios and the sensitivity (TP/(TP+FN)) are calculated for different LLR (log_2_LR) cutoffs. Each dot on the curves represents an average of ten cross-validations at a particular LLR cutoff. A. TP/FP ratios versus LLR cutoffs. A resolution of 44% false positives (TP/FP>1) corresponds to a LLR cutoff of 7.0 and 180,010 predicted interactions. Predicted interactions of higher confidence (larger TP/FP ratios) can be obtained by selecting LLR cutoffs higher than 7.0. B. TP/FP versus sensitivity. The TP/FP ratios indicate the accuracy at a certain resolution, while the sensitivity defines the ability of a test to detect true positives. Hence a tradeoff exists between accuracy and sensitivity of a prediction.

To evaluate the overall performance of the prediction, we did a ten-fold cross-validation. First we randomly divided both the GSP and GSN datasets into ten separate equal sets. Then we used nine of the ten sets as the training set to calculate the LRs and the remaining set as the test set to identify the positives and negatives. We ran this process ten times so that each of the ten sets was a test set and the remaining nine constituted the training set. Finally we summed up the number of true positives (TP), false positives (FP), true negatives (TN) and false negatives (FN) to get the sensitivity (TP/(TP+FN)) and TP/FP ratios under different LLR cutoffs (from 1 to 16). The TP/FP ratios are apparently correlated with LLR cutoffs (Figure 3A). They decrease with the increase in prediction sensitivity, indicating a tradeoff between the accuracy and sensitivity of predictions (Figure 3B). We set a LLR cutoff of 6.0 as the minimal requirement for a predicted PPI to be entered into IntNetDB, and 7.0 as the default prediction level because the TP/FP ratio is greater than 1 at this resolution. That is, at a LLR>7.0 we can get 56% of true positive protein-protein interactions within the 180,010 predicted protein-protein interactions among 9,901 proteins (Figure 3A).

### Improvements over the previous integration analysis for human PPI predictions

To obtain PPI predictions of higher quality and coverage, we used the latest HPRD dataset as the GSP dataset. HPRD now contains more than 10,000 newly annotated PPIs since the previous analysis of Rhodes *et al*. We integrated three more types of data (phenotype, genetic interactions and gene context) than used previously. For the interactome data type, we added three recently published interactome datasets [7,8,51], among which two are yeast two-hybrid screens using human proteins. For each new data type we have demonstrated the predictive power. We integrated two genetic interaction datasets, three gene context datasets [21,22] and three recently published phenotypic datasets of model organisms [52-54] (Table 1). The three more data types and the 14 extra datasets (more than double those in the previous analysis) integrated here make our analysis more information-rich, and also enabled us to predict 141,631 additional potential PPIs (4.7-fold increase in coverage) within a similar range of TP/FP ratio [27]. To facilitate use for researchers who are not familiar with network biology or bioinformatics, we have also implemented an easy-to-use and flexible data download, network visualization and analysis options online.

**Table 1:** Improvements over the previous integration analysis for human PPI predictions

		Rhodes *et al*.	This study
Gold standard positive (HPRD version)		Aug, 2004	Sep, 2005 (About 10000 more interactions)
Number of data types integrated		4 (PPI, GO, Microarray, DDI)	7 (PPI, GO, Microarray, DDI, Phenotypic, Genetic, Gene context)
Number of datasets integrated		13	27
Info from model organism		Only the PPI is applied	All the possible large scale data from model organism
Datasets for each data type	Gene expression	5	3
	PPI dataset	4 yeast, 1 worm and 1 fly	5 yeast, 1 worm, 2 fly and 2 Human
	GO dataset	Human	yeast, worm, fly, mouse and human
	Phenotypic	None	2 yeast and 1 fly datasets
	Genetic interaction	None	2 yeast datasets
	DDI interactions	Interpro annotation	Domain-domain interaction score dataset
	Gene context	None	3 yeast datasets
Method to validate the results		One simple test using the old HPRD as training set and the updated HPRD as test set	10-fold cross-validation
Size of integrated network under TP/FP ratio of 1		38,379 interactions among 5,791 proteins	180,010 interactions among 9,901 proteins
Data query		One time spread sheet download	Online query options with selectable data types and confidence level; data can be downloaded through spreadsheet or network graphs.
Network visualization and analysis		Only support one single gene's search and none for download or analysis	Network cluster extraction, visualization, drill-down and download options

## Utility

### Protein-protein interaction search

**Figure 4:**
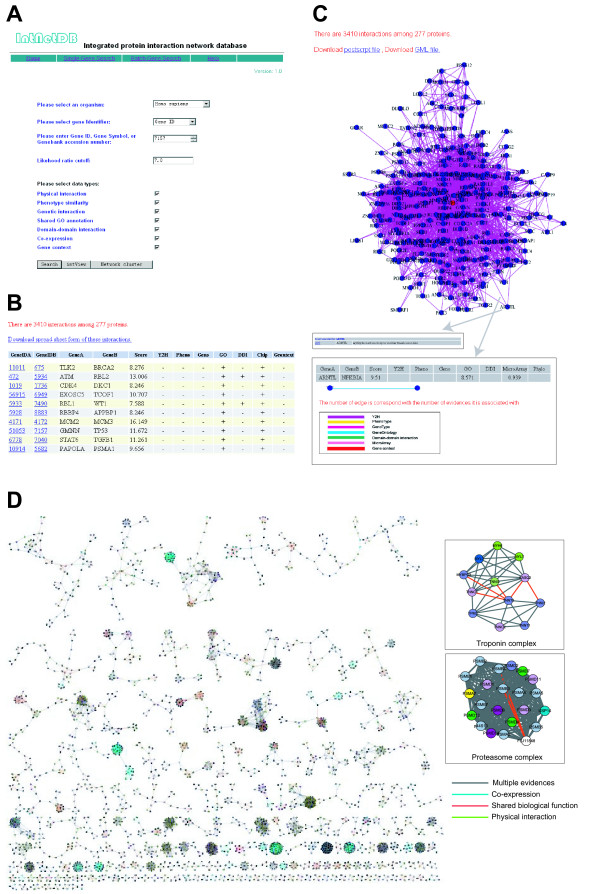
**Overview of IntNetDB**. A. The IntNetDB web interface. The default example is the network among human p53 (encoded by the TP53 gene) and its potential interaction partners. B. IntNetDB search results. For these query genes, the network graph view shows all predicted PPIs and the data types supporting each PPI, where '-' and '+' signs stand for 'absent' and 'present', respectively. A present call corresponds to LLR>0 for the data type shown. C. Visualization of the predicted PPI network. A hyperlink on the node and edge, when clicked, leads to more detailed information of the node or interaction (insets). D. Graphical representation of highly connected subgraphs. The subgraphs are extracted from the entire network by the MCODE algorithm. The two enlarged subgraphs correspond to the troponin-related complex and the proteasome complex. The color of an edge denotes the evidence type supporting the predicted PPI. 'Multiple evidences' are said to support a predicted interaction when more than one data type has LLR>1 (LR>2) for that edge. The color of a node is assigned according to the GO term of the gene.

IntNetDB provides a web-accessible query interface for users to search for potential PPIs among a list of query genes (Figure 4A). Query genes can be entered batch-wise using a variety of gene identifiers (NCBI Entrez GeneID, GeneSymbol, or GeneBank accession number) for different organisms (*C. elegans, D. melanogaster, M. musculus, and H. sapiens*). As yeast PPIs have been predicted comprehensively with various methods, we choose not to redo yeast PPI predictions with interolog information from other organisms, but rather refer readers to DIP, BIND and GRID PPI databases for this purpose [60-62], which are more comprehensive for yeast proteins. The purpose of this study was to generate a completely up-to-date PPI prediction database for higher organisms, information which is still lacking and greatly needed. For organisms other than human, no high quality PPI datasets are available to be used as a GSP (gold standard positive). Mapping by predicted interologs is the best that can be reached currently. The lack of a GSP also means it is not possible to estimate the accuracy of the predicted interologs. Therefore, instead of presenting model organism PPI as rigorous data mining conclusions, we provide information on worm, fly and mouse solely for reference and convenience of researchers. Other resources, such as Homologene [63] COG [64] and Inparanoid [65] may provide more precise ortholog information than what we provide here through reciprocal best hit method.

IntNetDB also provides information on LLR cutoffs. Users can obtain more reliable PPIs by selecting larger cutoff values, than the default value of 7.0. If users need as many predicted PPIs as possible for analysis, then the predicted PPIs with LLR>6.0 are also accessible from the website. Lower LLR includes more protein-protein interactions and proteins but of lower confidence. Confidence levels (percentage of expected true positive) of the predicted PPIs at different LLR cutoffs are also provided (Figure 3A** inset**). From the seven integrated heterogeneous data types currently available, users can select the exact data types that they want to access. A list of the PPIs under the user-defined LLR cutoff with data type information are generated after a gene list is submitted. The default gene list uses p53 and its interacting partners in human as an example. A tab-delimited text file of PPIs can be downloaded directly from the query result page (Figure 4B).

### Network visualization

Network visualization provides a more informative way than a simple textual list for exploration of a network. IntNetDB provides an online visualization tool for protein-protein interaction networks called 'intView'. A PPI network is represented as an undirected graph by intView, where nodes represent proteins and edges correspond to potential PPIs. Figure 4C shows the network layout of TP53 and its partners. Users can click the nodes or edges to see more information about them (Figure 4C** inset**). When an edge is clicked, a pop-up panel will display which data type(s) support the functional interaction between the two nodes. Different data types are denoted by different colors, and the width of the edge representing a data type is proportional to the LR value derived from that data type (Figure 4C** inset**). Online visualization is displayed with a scalable vector graphics (SVG) file. Postscript file and Cytoscape-compatible GML file can also be generated for download.

### Extracting highly connected modules

Densely interconnected neighborhoods or clusters in a network frequently correspond to functional modules [46,66]. IntNetDB provides a tool to extract and reveal such neighborhoods or clusters in the network of query genes. For this IntNetDB uses the MCODE algorithm [46], which was created by Bader *et al*. for predicting yeast protein complexes and implemented by Lee *et al *for automatically searching protein complexes in any organism [67]. The resulting modules can be visualized by clicking on the 'Network cluster' button on the web page. All the extracted clusters in IntNetDB are visualized in Cytoscape [68] (Figure 4D). For cross examination and fine control, the users are referred to a probabilistic model-based cluster-finding algorithm implemented in NetworkBlast [69].

An advantage of extracting such densely connected neighborhoods is that new functions can be assigned to a gene, or potential functions can be assigned to a gene of unknown function based on the functions of other genes in the same cluster [19]. As an example take the Troponin complex, a key protein complex regulating sarcomeric muscle contraction that is composed of three subunits, Tn-I, Tn-C and Tn-T. The Tn-I subunit inhibits actomyosin ATPase, while the Tn-T binds Tn-C and has high affinity for tropomyosin. With the release of intracellular calcium, Tn-C subunit binds calcium and the conformation of the troponin complex changes accordingly to overcome inhibition of actomyosin ATPase activity [70]. In the predicted human troponin complex, TNNI3 (coding for Tn-I subunit) directly interacts with CASQ1, a protein that binds and putatively stores calcium ions in the sarcoplasmic reticulum. This finding suggests that the Troponin complex may also regulate muscle contractions through direct association with intracellular calcium stores. The network modules also show the Tronponin complex tightly connected with structural proteins (MYH6, MYL3, MYL7, MYBPC3), suggesting that they may also function in the sarcomeric muscle contraction (Figure 4D** inset**).

Another example of an extracted functional module is the proteasome complex involved in ATP/ubiquitin-dependent peptide cleavage (Figure 4D** inset**). The proteasome is a multicatalytic proteinase complex comprised of many subunits. In the extracted network module a hypothetical protein, FLJ11848, is predicted to tightly interact with many subunits of the proteasome. Interestingly, FLJ11848 contains six WD40 domains, which are frequently found in adaptor or regulatory proteins. This observation suggests that FLJ11848 might have an important role in regulating the activity of the proteasome. This prediction has been validated by recent work demonstrating that FLJ11848 functions as a negative regulator of the proteasome by controlling the assembly/disassembly of the proteasome [71].

### Data and GUI updating scheme

To keep IntNetDB up to date, we will keep tracking newly published large-scale genomic and proteomic datasets, evaluate the performance of them for PPI prediction, and use them to update the IntNetDB. The addition of new datasets should increase the comprehensiveness of the human interactome. To allow for future extension, and to avoid the burden of updating the user interface each time new data are added, GUI items are dynamically generated from database entries to reflect the current data types available in IntNetDB

## Conclusion

We have integrated and evaluated potential PPIs from different up-to-date genomic and proteomic features. We have provided a user-friendly query and visualization platform which can be easily extended in the future when more data become available. The product of this effort, IntNetDB, will facilitate network and functional analysis.

## Methods

### Gold standard

The Human Protein Reference Database (HPRD) [47] is a manually annotated protein-protein interaction database with 19,438 distinct interactions among 5,983 proteins. As it is derived from the literature of high quality experimental results, we used it as the gold standard positive (GSP) dataset. We used the gold standard negative (GSN) dataset previously generated by Rhodes *et al *[27], which includes all possible pair-wise combinations between two sets of proteins that are assigned a subcellular localization of the plasma membrane (1,397 proteins) and the nucleus (2,224 proteins), respectively, by the Gene Ontology (GO) Consortium. The whole GSN set includes a total of 3,106,928 protein pairs. Ideally the GSP and GSN should have no overlapping interactions. Of the 19,438 protein pairs in the positive gold-standard, 4,863 protein pairs are both of known subcellular localization. Of these 4,863 protein pairs, there are 330 overlapping interactions (representing a fraction of 7% = 330/4,863). This is very small compared to the randomly expected size of the intersection (representing a fraction of 38% = 1856/4,863), which was computed by assigning the protein with the shuffled subcellular localization in the GSN set. Although the gold-standard sets are imperfect, they still can provide good approximations for PPI prediction.

### Datasets

HPRD dataset [47] was downloaded on November 13, 2005. GO annotations were downloaded from NCBI on March 10, 2005. The three recently published interactome datasets [7,8,51], the two genetic interaction datasets [21,22], the three gene context datasets [11] and the three recently published phenotypic datasets of the model organisms [52-54] were downloaded from the journal or authors' websites.

### Naïve Bayes model

We used the Naïve Bayes method described in Jansen *et al*. and Rhodes *et al*. [25,27]. We defined as positive when two proteins interact and as negative when they do not. Considering the total number of positive pairs within all the possible protein pairs, the prior odds of finding a positive pair is:

Oprior=P(positive)P(negative)
 MathType@MTEF@5@5@+=feaafiart1ev1aaatCvAUfKttLearuWrP9MDH5MBPbIqV92AaeXatLxBI9gBaebbnrfifHhDYfgasaacH8akY=wiFfYdH8Gipec8Eeeu0xXdbba9frFj0=OqFfea0dXdd9vqai=hGuQ8kuc9pgc9s8qqaq=dirpe0xb9q8qiLsFr0=vr0=vr0dc8meaabaqaciaacaGaaeqabaqabeGadaaakeaacqqGpbWtcqqGWbaCcqqGYbGCcqqGPbqAcqqGVbWBcqqGYbGCcqGH9aqpdaWcaaqaaiabdcfaqjabcIcaOiabdchaWjabd+gaVjabdohaZjabdMgaPjabdsha0jabdMgaPjabdAha2jabdwgaLjabcMcaPaqaaiabdcfaqjabcIcaOiabd6gaUjabdwgaLjabdEgaNjabdggaHjabdsha0jabdMgaPjabdAha2jabdwgaLjabcMcaPaaaaaa@51B4@

where P(positive) is the possibility of getting an interacting pair of proteins in all the possible interactions while P(negative) stands for the possibility of getting a pair of non-interacting proteins. In contrast, the posterior odds are the odds of getting a positive when we consider the given evidence:

Oposterior=P(positive|evidence1...evidenceN)P(negative|evidence1...evidenceN)
 MathType@MTEF@5@5@+=feaafiart1ev1aaatCvAUfKttLearuWrP9MDH5MBPbIqV92AaeXatLxBI9gBaebbnrfifHhDYfgasaacH8akY=wiFfYdH8Gipec8Eeeu0xXdbba9frFj0=OqFfea0dXdd9vqai=hGuQ8kuc9pgc9s8qqaq=dirpe0xb9q8qiLsFr0=vr0=vr0dc8meaabaqaciaacaGaaeqabaqabeGadaaakeaacqqGpbWtcqqGWbaCcqqGVbWBcqqGZbWCcqqG0baDcqqGLbqzcqqGYbGCcqqGPbqAcqqGVbWBcqqGYbGCcqGH9aqpdaWcaaqaaiabdcfaqjabcIcaOiabdchaWjabd+gaVjabdohaZjabdMgaPjabdsha0jabdMgaPjabdAha2jabdwgaLjabcYha8jabdwgaLjabdAha2jabdMgaPjabdsgaKjabdwgaLjabd6gaUjabdogaJjabdwgaLjabigdaXiabc6caUiabc6caUiabc6caUiabdwgaLjabdAha2jabdMgaPjabdsgaKjabdwgaLjabd6gaUjabdogaJjabdwgaLjabd6eaojabcMcaPaqaaiabdcfaqjabcIcaOiabd6gaUjabdwgaLjabdEgaNjabdggaHjabdsha0jabdMgaPjabdAha2jabdwgaLjabcYha8jabdwgaLjabdAha2jabdMgaPjabdsgaKjabdwgaLjabd6gaUjabdogaJjabdwgaLjabigdaXiabc6caUiabc6caUiabc6caUiabdwgaLjabdAha2jabdMgaPjabdsgaKjabdwgaLjabd6gaUjabdogaJjabdwgaLjabd6eaojabcMcaPaaaaaa@8F00@

where the evidence is a data type used to infer PPI between the proteins. The terms 'prior' and 'posterior' refer to the condition before and after considering the information provided by the N evidences. Then the likelihood ratio (L) is defined as:

L(evidence1...evidenceN)=P(evidence1...evidenceN|positive)P(evidence1...evidenceN|negative),
 MathType@MTEF@5@5@+=feaafiart1ev1aaatCvAUfKttLearuWrP9MDH5MBPbIqV92AaeXatLxBI9gBaebbnrfifHhDYfgasaacH8akY=wiFfYdH8Gipec8Eeeu0xXdbba9frFj0=OqFfea0dXdd9vqai=hGuQ8kuc9pgc9s8qqaq=dirpe0xb9q8qiLsFr0=vr0=vr0dc8meaabaqaciaacaGaaeqabaqabeGadaaakeaacqqGmbatcqGGOaakcqWGLbqzcqWG2bGDcqWGPbqAcqWGKbazcqWGLbqzcqWGUbGBcqWGJbWycqWGLbqzcqaIXaqmcqGGUaGlcqGGUaGlcqGGUaGlcqWGLbqzcqWG2bGDcqWGPbqAcqWGKbazcqWGLbqzcqWGUbGBcqWGJbWycqWGLbqzcqWGobGtcqGGPaqkcqGH9aqpdaWcaaqaaiabdcfaqjabcIcaOiabdwgaLjabdAha2jabdMgaPjabdsgaKjabdwgaLjabd6gaUjabdogaJjabdwgaLjabigdaXiabc6caUiabc6caUiabc6caUiabdwgaLjabdAha2jabdMgaPjabdsgaKjabdwgaLjabd6gaUjabdogaJjabdwgaLjabd6eaojabcYha8jabdchaWjabd+gaVjabdohaZjabdMgaPjabdsha0jabdMgaPjabdAha2jabdwgaLjabcMcaPaqaaiabdcfaqjabcIcaOiabdwgaLjabdAha2jabdMgaPjabdsgaKjabdwgaLjabd6gaUjabdogaJjabdwgaLjabigdaXiabc6caUiabc6caUiabc6caUiabdwgaLjabdAha2jabdMgaPjabdsgaKjabdwgaLjabd6gaUjabdogaJjabdwgaLjabd6eaojabcYha8jabd6gaUjabdwgaLjabdEgaNjabdggaHjabdsha0jabdMgaPjabdAha2jabdwgaLjabcMcaPaaacqGGSaalaaa@9F5C@

which relates prior and posterior odds according to the Bayes rule:

Oposterior = *L*(*evidence*1...*evidenceN*)*Oprior.

When N evidences are derived independently, the Bayes rule can be simplified to Naïve Bayes rule and L can be simplified as:

L(evidence1...evidenceN)=∏i=1NL(evidence i)=∏i=1NP(evidence1...evidenceN|positive)P(evidence1...evidenceN|negative)
 MathType@MTEF@5@5@+=feaafiart1ev1aaatCvAUfKttLearuWrP9MDH5MBPbIqV92AaeXatLxBI9gBaebbnrfifHhDYfgasaacH8akY=wiFfYdH8Gipec8Eeeu0xXdbba9frFj0=OqFfea0dXdd9vqai=hGuQ8kuc9pgc9s8qqaq=dirpe0xb9q8qiLsFr0=vr0=vr0dc8meaabaqaciaacaGaaeqabaqabeGadaaakeaacqqGmbatcqGGOaakcqWGLbqzcqWG2bGDcqWGPbqAcqWGKbazcqWGLbqzcqWGUbGBcqWGJbWycqWGLbqzcqaIXaqmcqGGUaGlcqGGUaGlcqGGUaGlcqWGLbqzcqWG2bGDcqWGPbqAcqWGKbazcqWGLbqzcqWGUbGBcqWGJbWycqWGLbqzcqWGobGtcqGGPaqkcqGH9aqpdaqeWbqaaiabdYeamjabcIcaOiabdwgaLjabdAha2jabdMgaPjabdsgaKjabdwgaLjabd6gaUjabdogaJjabdwgaLjabbccaGiabdMgaPjabcMcaPaWcbaGaemyAaKMaeyypa0JaeGymaedabaGaemOta4eaniabg+GivdGccqGH9aqpdaqeWbqaamaalaaabaGaemiuaaLaeiikaGIaemyzauMaemODayNaemyAaKMaemizaqMaemyzauMaemOBa4Maem4yamMaemyzauMaeGymaeJaeiOla4IaeiOla4IaeiOla4IaemyzauMaemODayNaemyAaKMaemizaqMaemyzauMaemOBa4Maem4yamMaemyzauMaemOta4KaeiiFaWNaemiCaaNaem4Ba8Maem4CamNaemyAaKMaemiDaqNaemyAaKMaemODayNaemyzauMaeiykaKcabaGaemiuaaLaeiikaGIaemyzauMaemODayNaemyAaKMaemizaqMaemyzauMaemOBa4Maem4yamMaemyzauMaeGymaeJaeiOla4IaeiOla4IaeiOla4IaemyzauMaemODayNaemyAaKMaemizaqMaemyzauMaemOBa4Maem4yamMaemyzauMaemOta4KaeiiFaWNaemOBa4MaemyzauMaem4zaCMaemyyaeMaemiDaqNaemyAaKMaemODayNaemyzauMaeiykaKcaaaWcbaGaemyAaKMaeyypa0JaeGymaedabaGaemOta4eaniabg+Givdaaaa@BC9D@

The Likelihood ratio (L) of evidence can be calculated from the positive and negative hits by binning all the evidences into discrete intervals. Then the integrated L can be multiplied from all the independent evidences.

### Orthologs

Human orthologs in mouse, fly, worm and yeast were identified as the best reciprocal BlastP hits with e-value cutoff of 10^-6 ^based on RefSeq protein sequences downloaded on December 9, 2004.

## Availability and requirements

The web interface of IntNetDB is freely accessible at . The graphical layouts are based on SVG, which requires Mozilla version 1.8 and up or installation of SVGviewer for Internet Explorer.

## Authors' contributions

J.-D.J.H. conceived and directed the project, designed the analyses, and analyzed data. K.X. and D.D. implemented the analyses, constructed the database and developed the website. J.-D.J.H., K.X. and D.D. wrote the paper. All authors read and approved the final manuscript.
